# Assessment of healthcare workers knowledge and attitudes towards Mpox infection at University of Gondar Comprehensive Specialized Referral Hospital, Ethiopia

**DOI:** 10.3389/fpubh.2025.1527315

**Published:** 2025-03-03

**Authors:** Alemante Tafese Beyna, Ermias Teklehaimanot Yefter, Assefa Belay Asrie, Habtamu Semagne Ayele, Tafere Mulaw Belete, Wondim Ayenew, Gashaw Sisay Chanie, Liknaw Workie Limenh, Melese Legesse Mitku, Mihret Melese, Gizachew Kassahun Bizuneh, Assefa Kebad Mengesha

**Affiliations:** ^1^Department of Pharmacology, School of Pharmacy, College of Medicine and Health Sciences, University of Gondar, Gondar, Ethiopia; ^2^Department of Pathology, School of Medicine, College of Medicine and Health Sciences, University of Gondar, Gondar, Ethiopia; ^3^Department of Social and Administrative Pharmacy, School of Pharmacy, College of Medicine and Health Sciences, University of Gondar, Gondar, Ethiopia; ^4^Department of Clinical Pharmacy, School of Pharmacy, College of Medicine and Health Sciences, University of Gondar, Gondar, Ethiopia; ^5^Department of Pharmaceutics, School of Pharmacy, College of Medicine and Health Sciences, University of Gondar, Gondar, Ethiopia; ^6^Department of Pharmaceutical Chemistry, School of Pharmacy, College of Medicine and Health Sciences, University of Gondar, Gondar, Ethiopia; ^7^Department of Human Physiology, School of Medicine, College of Medicine and Health Sciences, University of Gondar, Gondar, Ethiopia; ^8^Department of Pharmacognosy, School of Pharmacy, College of Medicine and Health Sciences, University of Gondar, Gondar, Ethiopia

**Keywords:** Mpox, healthcare workers, knowledge, attitudes, preparedness

## Abstract

**Background:**

Healthcare workers are on the frontlines of fighting health risks, especially during epidemics. Despite their critical role, their knowledge and attitudes toward Mpox have not been previously evaluated at the University of Gondar Comprehensive Specialized Hospital, Ethiopia. This study aimed to assess the knowledge and attitudes of healthcare workers regarding Mpox at this hospital.

**Method:**

This cross-sectional study assessing 382 HCWs. It was conducted on August 1–30, 2024. Data was collected using a self-administered questionnaire. A simple random sampling technique was used. Bivariate and multivariate binary logistic regression analysis was used. *p* < 0.05 was considered as Significant.

**Result:**

Almost half of the participants (48.40%) showed good knowledge, and 49.20% displayed a positive attitude toward Mpox. Age ≥ 50 Years old (AOR = 4.1, 95% CI 1.33–12.07), Physicians (AOR = 3.2, 95% CI 1.57–6.50), pharmacists (AOR = 3.5, 95% CI 1.55–8.06), having a B.Sc. Degree (AOR = 3.2, 95% CI 1.58–6.84), having M.Sc. (AOR = 3.3, 95% CI 1.60–6.84), work experience of 5–10 years (AOR = 3.2, 95% CI 1.78–5.95), HCWs who get information primarily from training (AOR = 2.7, 95% CI 1.08–6.70), and HCWs attending training including Mpox (AOR = 1.9, 95% CI 1.16–3.19) were more likely to having good knowledge than their counter. HCWs those having a M.Sc. degree (AOR = 2.1, 95% CI 1.11–4.20), physician (AOR = 2.1, 95% CI 1.10–4.16), pharmacist (AOR = 2.6, 95% CI 1.21–5.76), HCWs having work experience of 5–10 (AOR = 2.5, 95% CI 1.44–4.38), and HCWs attending training including Mpox (AOR = 1.9, 95% CI 1.18–3.07) were more likely to have positive attitude than their counter.

**Conclusion:**

This study revealed nearly half of the participants had a limited knowledge and negative attitudes. To addressing this gap it is essential to support training, integrate education, and improve resource accessibility.

## 1 Background

Mpox is a zoonotic virus that can infect animals and human (1). Scientists first spotted it in 1958 in a group of captive cynomolgus macaques (*Macaca fascicularis*) (2). This double-stranded DNA virus causes the disease Mpox, which is now spreading faster around the world (3). This virus belongs to the Orthopoxvirus genus and the Poxviridae family, which also includes viruses like smallpox and cowpox (4). Over time, the Mpox virus has evolved, becoming better adapted to surviving in new environments and infecting various animal species. People can catch the virus from animals, raising concerns that future viral strains may spread more easily or cause more severe disease in humans (5).

In 1958, the Mpox virus was first identified in monkeys in Copenhagen, Denmark (6). Mpox was first observed in humans in 1970 in the Democratic Republic of the Congo (7). Cases of Mpox have mostly been endemic in West and Central Africa (8). Since 1970, human Mpox cases have been reported in 11 African countries, including the Democratic Republic of the Congo, Benin, South Sudan, Gabon, Liberia, Cameroon, Nigeria, Côte d’Ivoire, Sierra Leone, and Central African Republic (9). The Mpox outbreak among humans has raised widespread alarm in numerous nations outside of Africa (10). For the first time, in 2003, cases of Mpox were reported outside of Africa in the USA, the UK in 2018, Singapore in 2019, and Israel in 2021 (11, 12). It is the first time in recorded human history; that epidemics of Mpox are spreading over the world. Considering the atypically vast scope and rapid dissemination in non-endemic nations (13).

Currently, there have been reports of Mpox virus epidemic in several nations on almost every continent (14). In contrast to isolated instances connected to visits to endemic areas, the precise source of the present infections is yet unknown (15). According to the CDC, by September 16, 2024, Mpox had led to over 100,000 cases across 122 countries. This includes 115 countries where people had not seen the Mpox virus before (16). The epidemic’s extraordinarily frequent rate of human-to-human transfer raises questions about the disease’s origins and mode of transmission. The primary way that Mpox is transmitted from person to person is through intimate contact with an infected individual (17). Additionally, if someone with Mpox has touched certain items, including clothing, bedding, towels, electronics, and surfaces, the virus may linger there for a while. It could also be transmitted to anyone who comes into contact with these objects. Further, It can also disseminate mother-to-fetus and sexual activity (18).

Mpox symptoms can be mild or serious. The main sign is a rash that lasts 2–4 weeks often with fever, body aches, and swollen glands (19). The rash shows up as blisters on different parts of the body, including the genitals and mouth (20). People can have anywhere from one to thousands of lesions. Some folks get inflammation in their rectum or genitals which can lead to pain or problems peeing. Mpox symptoms usually resolve within weeks with basic care. However, some cases can be severe and lead to complications or even death (21). The management of Mpox relies on the symptoms and prevents prolonged impact (19). Recent advancements in deep learning models, such as the InceptionV3-based approach, have demonstrated exceptional promise in image-based diagnostics, achieving high accuracy in Monkeypox detection (22). Certain drugs developed against smallpox have generated results that could be useful for Mpox (23). One such drug is Tecovirimat, approved for Mpox in Europe during outbreaks, but, still in the study with the aim of further improving its future use (24). Currently, the WHO recommends the use of MVA-BN or LC16 vaccines, or the ACAM2000. The infection of human Mpox is controlled by basic public health measures: personal protective equipment (PPE), hand hygiene, isolation, and contact tracing, as well as avoidance of infected animals. Healthcare workers should wear proper PPE including gloves and N95 masks when working with suspected cases (25).

Healthcare workers (HCWs) must be well-prepared because they are on the front lines of the Mpox epidemic. It’s challenging to predict how they will respond to a new danger such as Mpox given the continuing COVID-19 pandemic. Understanding how HCWs are aware of and prepared for this outbreak is essential for developing public health initiatives and influencing health legislation (26). Ethiopia is at an increased risk of Mpox infection due to the shared border with Sudan and Somalia, where cases have been reported (27). The other aggravating factor is the ongoing political instability in the region further complicates the delivery of effective healthcare services to the community. This study is unique because it focuses on a specialized hospital near the border, an area facing distinct health challenges. It seeks to provide meaningful insights into healthcare workers’ knowledge and attitudes toward Mpox, contributing valuable information to shape public health strategies and strengthen healthcare responses in high-risk regions. This study is designed to evaluate the knowledge and attitudes of healthcare workers regarding Mpox at the Gondar University Comprehensive Specialized Referral Hospital, Ethiopia.

## 2 Methods

### 2.1 Study design and setting

This cross-sectional study was carried out between August 1–30, 2024, among healthcare professionals at the University of Gondar Comprehensive Specialized Referral Hospital. This hospital is located in Gondar town, which is approximately 748 km far from Addis Ababa, the capital city of Ethiopia. This is one of a long-serving healthcare facility that acts as a multidisciplinary teaching referral hospital and thus has become an important health service provider for a population of more than 7 million people living in the northwestern part of the country. It provides a wide range of medical services and training opportunities for healthcare professionals. The hospital employs a total of 2,331 staff members, including 1,098 health professionals and 217 medical doctors. The hospital is approximately 190 km away from Metema, a populous border town that is an important route to Sudan, thus adding value to its accessibility and importance for the service provision of healthcare for both local and cross-border populations.

### 2.2 Source and study population

The source and study population comprised all healthcare workers working in Gondar University Comprehensive Specialized Referral Hospital. This included health professionals from diverse fields such as medicine, nursing, pharmacy, laboratory services, midwifery, and diagnostic units, all of whom work collaboratively across different departments.

### 2.3 Inclusion and exclusion criteria

This study included all healthcare workers at the Gondar University Comprehensive Specialized Referral Hospital. Certain medical practitioners who were unavailable for data collection or who were on maternity or annual leave were not included in the study.

### 2.4 Sample size calculation and sampling technique

To determine the sample size, the single population proportion formula was applied. The sample size was determined using the proportion of knowledge level of Health workers from the previous study. The proportions were 38.5% (28).


$$n=\frac{{\left(Z\alpha /2\right)}^{2}\times P\left(1-P\right)}{d^{2}}$$

$$n=\frac{{\left(1.96\right)}^{2}\times 0.385\;\left(1–0.385\right)}{0.05^{2}}$$

$$n=\frac{3.84\times 0.385\;\left(0.615\right)}{0.0025}=363.8$$

Where, p = proportion, n = calculated sample size, (*α* = 0.05), 95% confidence interval (Z α/2 = 1.96), and absolute precision or margin of error, 5% (d = 0.05). The total sample size for Health workers was 363.8. By adding a 10% non-response rate a 364*10% = 36.4, the final sample size was 401. A total of 401 healthcare workers were selected to participate in the study using a simple random sampling procedure from the 1,315 healthcare workers employed at Gondar University Comprehensive Specialized Referral Hospital.

### 2.5 Data collection tools and procedures

A standardized, self-administered questionnaire was used to collect data. The data collection instrument was adopted from previous research (29–31). Data were collected by three trained data collectors and one supervisor. Prior to the start of the data collection, Gondar University Comprehensive Specialized Referral Hospital participant healthcare workers gave their written informed consent. The questionnaire’s first portion contains Socio-demographic characteristics and Professional Experience, and the subsequent sections evaluated HCWs knowledge and attitudes towards Mpox infections. The demographic factors encompassed age, sex, occupational, educational status, and work experience. Healthcare worker’s Mpox knowledge was assessed using a 17-item comprehensive question. The question item was designed based on insights from previous studies. Participants had three possible answers to choose from, “yes,” “no,” or “I do not know.” This questionnaire assesses individual general knowledge, route of transmission, clinical presentation, vulnerable group, and case management of Mpox infection. Each correct answer received a score of 1, while wrong answers and “I do not know” received a score of 0. The overall knowledge score, which ranges from 0 to 17, was calculated by adding the scores together. A higher number denotes greater knowledge. The attitude of healthcare workers was assessed using 9 items questions. Participants had a 5-Likert scale of possible answers to choose. Which included options ranging from: “strongly disagree,” “disagree,” “neutral,” “agree,” and “strongly agree.” Each positive attitude question received a score of 1 for “strongly disagree,” 2 for “disagree,” 3 for “neutral,” 4 for “agree,” and 5 for “strongly agree.” To maintain consistency with positively framed statements, any negative questions were scored in reverse to align appropriately. The overall attitude score, which ranges from 9 to 45, was calculated by adding the scores together. A higher number denotes a positive attitude towards Mpox. Healthcare workers were classified as having good knowledge if their score was above the mean of 8.24, and as having poor knowledge if their score was below or equal to the mean. In a similar vein, healthcare professionals were deemed to have a positive attitude if their score was higher than the mean of 28.49, and a negative attitude if their score was lower or equal to the mean.

### 2.6 Data quality control

Intensive training was given to allocate data collector and supervisor. The training covered data collection processes, emphasizing clarity on terms and tools, and study objectives. It also stressed timely organization and submission of collected data. The data collection tool was pretested on 5% (20) of a health worker that was not included in the final analysis to validate the consistency of the questions and data collection tool. Based on the findings, some changes were made, such as rewording and adjusting the data collection tool. The principal investigator and the supervisor did the daily follow-up throughout data collection. Daily completeness of each questionnaire was checked by the supervisor and the principal investigators.

### 2.7 Statistical analysis

The collected data were cleared, coded, entered into Epi-info version 7, and analyzed using SPSS version 25.0. The results are described in terms of frequencies and percentages. The data were showcased through descriptive texts, tables, and figures. The baseline characteristics regarding knowledge and attitude of Mpox were compared by Using Pearson’s Chi-square (*χ*^2^) test. Both bivariate and multivariate logistic regression were used to identify the most influential predictors of Mpox infection knowledge and attitude of HCWs. Variables with a *p* value less than 0.25 in the bivariate analysis were included in multivariate logistic regression models. *p* < 0.05 was considered statistically significant.

### 2.8 Ethical considerations

A formal letter of approval was obtained from the Ethical Review Board of the School of Pharmacy, College of Medicine and Health Science, University of Gondar, with protocol number SOP 087/10/11/2016. This research was carried out following the principles outlined in the Declaration of Helsinki, ensuring ethical standards were met. Each participant in the study was informed about confidentiality. Each participant expresses their willingness to participate in this study, which is approved by their written consent. Participants were allowed to discontinue the research at any time.

## 3 Result

### 3.1 Socio-demographic characteristics

The questionnaire was completed by 382 out of 401 participants, yielding a 95.3% response rate. The participant’s ages ranged from 22 to 59 years old, with a mean age of 34.5 (SD ± 7.70). The majority of participants were male (224, 58.6%), held a B.Sc. degree (160, 41.9%), and had working experience of 5–10 years (164, 42.9%). In the comparison of various professions, nurses constituted the largest group, numbering 112 (29.3%), followed by physicians at 71 (18.6%), midwives at 62 (16.2%), and pharmacists at 51 (13.4%) (Table 1).

**Table 1:** Socio-demographic characteristics and professional experience of participant HCWs at Gondar University Comprehensive Specialized Referral Hospital, northwest Ethiopia 2024 (*N* = 382).

Variable		Frequency (%)
Age	<35 Years	210 (55.0)
	35–50 Years	143 (37.4)
	≥50 Years	29 (7.6)
Gender	Male	224 (58.6)
	Female	158 (41.4)
Occupation	Nurse	112 (29.3)
	Physician	71 (18.6)
	Pharmacist	51 (13.4)
	Laboratory technologist	46 (12.0)
	Midwives	62 (16.2)
	Other (Radiologist, physiotherapists…)	40 (10.5)
Educational status	Diploma or certificate	70 (18.3)
	B.Sc. Degree	160 (41.9)
	M.Sc. and above	152 (39.8)
Work experience	<5 Years	115 (30.1)
	5–10 Years	164 (42.9)
	≥10 Years	103 (27)
Where do you primary get your information on Mpox?	Colleagues	55 (14.4)
	Medical book or during study	118 (30.9)
	Training/workshop	53 (13.9)
	Social media	81 (21.2)
	Main stream media	75 (19.6)
Have you received any specific training related to infectious disease (including Mpox)?	No	180 (47.1)
	Yes	202 (52.9)
Have you had any direct experience managing or treating patients with infectious diseases (e.g., COVID-19, smallpox, etc.)?	No	150 (39.3)
	Yes	232 (60.7)

### 3.2 Healthcare workers knowledge regarding Mpox

**Figure 1:**
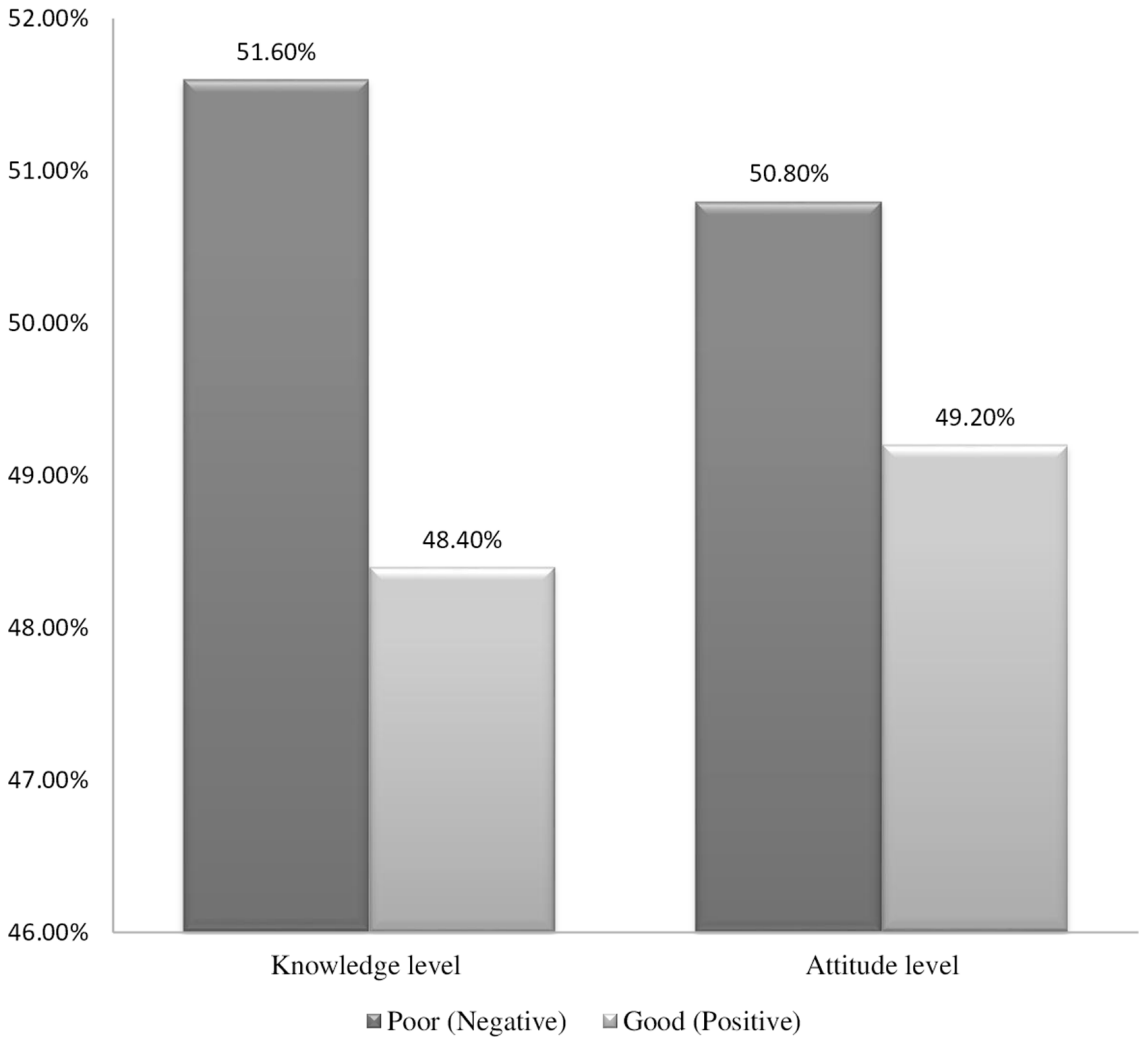
Knowledge and attitude of Mpox study participants working at the Gondar University Comprehensive Specialized Referral Hospital, northwest Ethiopia 2024 (*N* = 382).

Among all HCWs participants, 48.40% of participants showed good Mpox knowledge, scoring a mean of 8.24 (SD ± 2.70) out of 17 (Figure 1). According to the knowledge evaluation, the majority of respondents 205 (53.7%) know Mpox is prevalent in Western and Central Africa, while 297 (77.7%) believe it is caused by a virus. Furthermore, the majority of HCWs 220 (57.6), 189 (49.5), and 208 (54.5) understand Young children, Pregnant women, and Immune-compromised patients, respectively, are at increased risk for severe Mpox disease (Table 2).

**Table 2:** Description of the knowledge scale items’ responses toward Mpox among HCWs at the Gondar University Comprehensive Specialized Referral Hospital, northwest Ethiopia 2024 (*N* = 382).

	Items	“Yes” (%)	“Do not know” (%)	“No” (%)
1	Mpox is prevalent in the Middle East	164 (42.9)	143 (37.4)	**75 (19.6)**
2	Mpox is prevalent in Western and Central Africa	**205 (53.7)**	116 (30.3)	61 (16.0)
3	There is an outbreak of human Mpox in the world	**217 (56.8)**	82 (21.5)	83 (21.7)
4	Mpox is caused by a virus	**297 (77.7)**	43 (11.3)	42 (11.0)
5	Human-to-human transmission of Mpox occurs through skin-to-skin contact	**208 (54.5)**	93 (24.3)	81 (21.2)
6	Human-to-human transmission of Mpox occurs through touching objects or surfaces that have been used by someone with Mpox	**185 (48.4)**	121 (31.7)	76 (19.9)
7.	Human-to-human transmission of Mpox occurs through contact with respiratory secretions	**165 (43.2)**	167 (43.7)	50 (13.1)
8.	Mpox and smallpox have similar signs and symptoms	**182 (47.6)**	132 (34.6)	68 (17.8)
9.	Skin rash is one of the signs or symptoms of human Mpox	**277 (72.5)**	78 (20.4)	27 (7.1)
10	Pustule is one of the signs or symptoms of human Mpox	**189 (49.5)**	144 (37.7)	49 (12.8)
11.	Antibiotics are used to treat human Mpox	93 (24.3)	141 (36.9)	**148 (38.7)**
12.	Diarrhea is one of the signs or symptoms of human Mpox	140 (36.6)	153 (40.1)	**89 (23.3)**
13.	Vaccination is available to prevent human Mpox	**167 (43.7)**	134 (35.1)	21.2 (81)
14	Young children less than 8 years of age are at increased risk for severe Mpox disease	**220 (57.6)**	111 (29.1)	51 (13.4)
15.	Pregnant women are at increased risk for severe Mpox disease	**189 (49.5)**	137 (35.9)	56 (14.7)
16.	Immune-compromised patients are at increased risk for severe Mpox disease	**208 (54.5)**	126 (33.0)	48 (12.6)
17.	Individuals with a history of atopic dermatitis or eczema are at increased risk for severe Mpox disease	**130 (34.0)**	191 (50.0)	61 (16.0)

Numbers in bold indicate correct responses.

### 3.3 Factors associated with healthcare workers knowledge about Mpox

Bivariate and multivariate logistic regression analyses were conducted to assess the association between HCW knowledge of Mpox and independent variables. In the bivariate analysis, age, occupation, educational status, work experience, source of information, training related to infectious disease, and experience in managing infectious disease were selected variables with a *p*-value <0.25 for multivariate regression analysis. In multivariate regression, age, occupation, educational status, work experience, source of information, and training related to infectious disease were significantly associated factors (Table 3). This indicates that being ≥50 Years old was 4.1 (AOR = 4.1, 95% CI 1.33–12.07) times more likely to have good knowledge of Mpox than younger. Similarly, Physicians and pharmacists were 3.2 (AOR = 3.2, 95% CI 1.57–6.50) and 3.5 (AOR = 3.5, 95% CI 1.55–8.06) times more likely, respectively, to possess a strong understanding of Mpox compared to nurses. Other associated factors are educational status, having a B.Sc. Degree and M.Sc. and above were 3.2 (AOR = 3.2, 95% CI 1.58–6.84) and 3.3 (AOR = 3.3, 95% CI 1.60–6.84) times more likely, respectively, to have a good knowledge than HCWs having Diploma or Certificate. Correspondingly, those having work experience of 5–10 years were 3.2 (AOR = 3.2, 95% CI 1.78–5.95) times more likely to have good knowledge of Mpox compared to those with less experience HCWs. In addition to this, HCWs who get information primarily from training and attending training including Mpox were 2.7 (AOR = 2.7, 95% CI 1.08–6.70) and 1.9(AOR = 1.9, 95% CI 1.16–3.19) times more likely, respectively, to have a good knowledge than those HCWs get information to colleagues and not receive any training (Table 3).

**Table 3:** Multivariable logistic regression analysis on factors associated with knowledge toward Mpox among HCWs at the Gondar University Comprehensive Specialized Referral Hospital, northwest Ethiopia 2024 (*N* = 382).

Variable		Knowledge level		COR (95% CI)	*p*	AOR (95% CI)	*p*
		Good (%)	Poor (%)				
Age	<35 Years	86 (41)	124 (59)	1	1	1	
	35–50 Years	79 (55.2)	64 (44.8)	1.8 (1.16–2.73)	0.008	1.4 (0.89–2.47)	0.131
	≥50 Years	20 (69)	9 (31)	3.2 (1.39–7.37)	0.006	4.1 (1.33–12.07)	0.013^*^
Occupation	Nurse	46 (41.1)	66 (58.9)	1	1	1	
	Physician	47 (66.2)	24 (33.8)	2.8 (1.51–5.21)	0.001	3.2 (1.57–6.50)	0.001^*^
	Pharmacist	32 (62.4)	19 (37.3)	2.4 (1.22–4.77)	0.011	3.5 (1.55–8.06)	0.003^*^
	Laboratory technologist	18 (39.1)	28 (60.9)	0.9 (0.45–1.86)	0.821	1.1 (0.46–2.61)	0.834
	Midwives	30 (48.4)	32 (51.6)	1.3 (0.72–2.51)	0.352	1.7 (0.81–3.54)	0.158
	Other (radiologist, physiotherapists…)	12 (30)	28 (70)	0.6 (0.28–1.34)	0.218	1.1 (0.42–2.83)	0.846
Educational status	Diploma or certificate	20 (28.6)	50 (71.4)	1	1	1	
	B.Sc. Degree	81 (50.6)	79 (49.4)	2.5 (1.40–4.69)	0.002	3.2 (1.58–6.84)	0.001^*^
	M.Sc. and above	84 (55.3)	68 (44.7)	3.1 (1.67–5.67)	0.000	3.3 (1.60–6.84)	0.001^*^
Work experience	<5 Years	34 (29.6)	81 (70.4)		1	1	
	5–10 Years	94 (57.3)	70 (42.7)	3.1 (1.92–5.30)	0.000	3.2 (1.78–5.95)	0.000^*^
	≥10 Years	57 (55.3)	46 (44.7)	2.9 (1.69–5.15)	0.000	2.6 (1.33–5.15)	0.005
Where do you primary get your information on Mpox?	Colleagues	28 (50.9)	27 (49.1)	1	1	1	
	Medical book or during study	39 (33.1)	79 (66.9)	0.41 (0.24–0.91)	0.026	0.7 (0.33–1.51)	0.375
	Training/workshop	29 (54.7)	24 (45.3)	1.1 (0.54–2.48)	0.692	2.7 (1.08–6.70)	0.032^*^
	Social media	46 (56.8)	35 (43.2)	1.2 (0.63–2.52)	0.499	2.1 (0.92–4.73)	0.077
	Main stream media	43 (57.3)	32 (42.7)	1.2 (0.64–2.61)	0.468	2.2 (0.97–5.26)	0.057
Have you received any specific training related to infectious disease (including Mpox)?	No	71 (39.4)	109 (60.6)	1	1	1	
	Yes	114 (56.4)	88 (43.6)	1.9 (1.32–2.99)	0.001	1.9 (1.16–3.19)	0.011^*^
Have you had any direct experience managing or treating patients with infectious diseases (e.g. COVID-19, smallpox, etc.)?	No	59 (39.3)	91 (60.7)	1	1	1	
	Yes	126 (54.3)	106 (45.7)	1.8 (1.21–2.78)	0.004	1.3 (0.81–2.38)	0.238

*Significant at *p* < 0.05.

### 3.4 Healthcare workers attitude regarding Mpox

**Figure 2:**
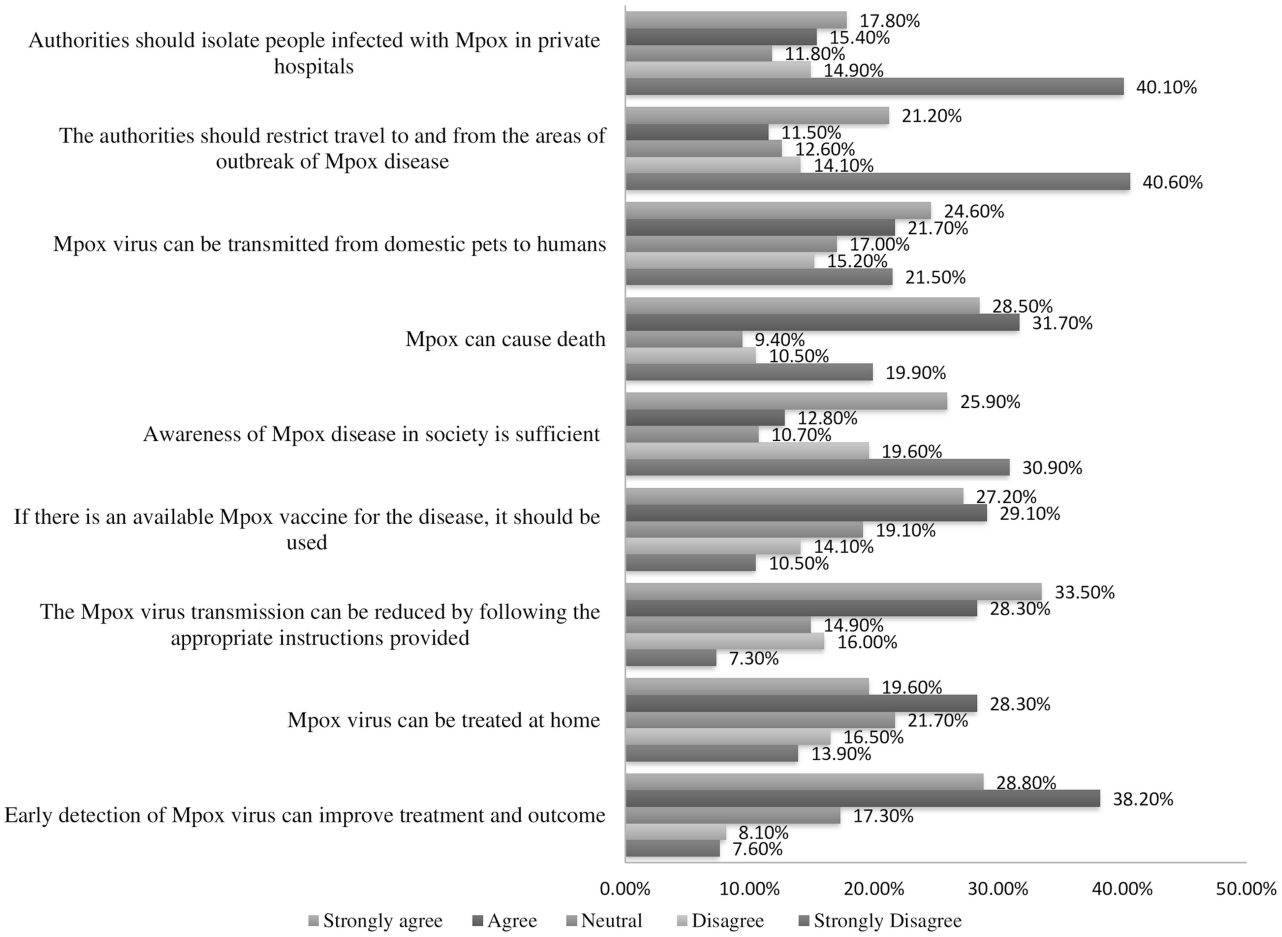
Description of the attitude scale items’ responses toward Mpox among healthcare workers at the Gondar University Comprehensive Specialized Referral Hospital, northwest Ethiopia 2024 (*N* = 382).

Of the 382 participants, 49.20% showed a positive attitude for Mpox, scoring a mean of 28.49 (SD ± 4.35) out of 45 (Figure 1). According to the attitude scale question, 38.2% of the participants agreed that early detection of the Mpox virus can improve treatment and outcome. Likely, 31.7% of respondents agreed Mpox can cause death. In oppose to this, 25.9% of them believe (strongly agree) awareness of the Mpox disease in society is sufficient. Correspondingly, 28.3% of participants agree Mpox virus can be treated at home (Figure 2).

### 3.5 Factors associated with healthcare worker’s attitude to Mpox

In bivariate analysis, age, occupation, educational status, work experience, source of information, training related to infectious disease, and experience in managing infectious disease were selected variables with a *p*-value <0.25 for multivariate regression analysis. In multivariate regression, occupation, educational status, work experience, and training related to infectious disease were significantly associated factors. This indicates that being a physician was associated with a 2.1 times higher likelihood of having a positive attitude of Mpox compared to nurse (AOR = 2.1, 95% CI 1.10–4.16) while being a pharmacist was associated with a 2.6 times higher likelihood of having a positive attitude of Mpox (AOR = 2.6, 95% CI 1.21–5.76). Similarly, those having a M.Sc. degree and above were 2.1 (AOR = 2.1, 95% CI 1.11–4.20) times more likely, to have a positive attitude than HCWs having a Diploma or Certificate. In the same way, those having work experience of 5–10 years were 2.5 times more likely to have a positive attitude of Mpox compared to those with less experience HCWs (AOR = 2.5, 95% CI 1.44–4.38). Correspondingly, HCWs attending training including Mpox were 1.9 (AOR = 1.9, 95% CI 1.18–3.07) times more likely to have a positive attitude than those not receive any training (Table 4).

**Table 4:** Multivariable logistic regression analysis on factors associated with attitude toward Mpox among HCWs at the Gondar University Comprehensive Specialized Referral Hospital, northwest Ethiopia 2024 (*N* = 382).

Variable		Attitude level		COR (95% CI)	*p*	AOR (95% CI)	*p*
		Positive (%)	Negative (%)				
Age	<35 Years	90 (42.9)	120 (57.1)	1	1	1	
	35–50 Years	79 (55.2)	64 (44.8)	1.6 (1.07–2.52)	0.023	1.4 (0.87–2.29)	0.161
	≥50 Years	19 (65.5)	10 (34.5)	2.5 (1.12–5.71)	0.025	2.6 (0.99–7.14)	0.052
Occupation	Nurse	47 (42.0)	65 (58.0)	1	1	1	
	Physician	42 (59.2)	29 (40.8)	2.0 (1.09–3.66)	0.024	2.1 (1.10–4.16)	0.025^*^
	Pharmacist	30 (58.8)	21 (41.2)	1.9 (1.01–3.86)	0.047	2.6 (1.21–5.76)	0.015^*^
	Laboratory technologist	23 (50)	23 (50)	1.3 (0.69–2.75)	0.356	1.8 (0.81–4.22)	0.140
	Midwives	30 (48.4)	32 (51.6)	1.2 (0.69–2.41)	0.414	1.7 (0.85–3.53)	0.124
	Other (radiologist, physiotherapists…)	16 (40.0)	24 (60.0)	0.92 (0.44–1.92)	0.829	1.7 (0.71–4.12)	0.227
Educational status	Diploma or certificate	25 (35.7)	45 (64.3)	1	1	1	
	B.Sc. Degree	76 (47.5)	84 (52.5)	1.6 (0.91–2.90)	0.099	1.8 (0.92–3.50)	0.084
	M.Sc. and above	87 (57.2)	65 (42.8)	2.4 (1.34–4.32)	0.003	2.1 (1.11–4.20)	0.023^*^
Work experience	<5 Years	38 (33.0)	77 (67.0)	1	1	1	
	5–10 Years	93 (56.7)	71 (43.3)	2.6 (1.61–4.36)	0.000	2.5 (1.44–4.38)	0.001^*^
	≥10 Years	57 (55.3)	46 (44.7)	2.5 (1.44–4.35)	0.001	1.7 (0.94–3.27)	0.077
Where do you primary get your information on Mpox?	Colleagues	30 (54.5)	25 (45.5)	1	1	1	
	Medical book or during study	45 (38.1)	73 (61.9)	0.5 (0.26–0.98)	0.044	0.6 (0.29–1.27)	0.187
	Training/workshop	29 (54.7)	24 (45.3)	1.0 (0.47–2.14)	0.986	1.4 (0.61–3.49)	0.382
	Social media	42 (51.9)	39 (48.1)	0.8 (0.45–1.78)	0.757	1.1 (0.51–2.45)	0.761
	Main stream media	42 (56.0)	33 (44.0)	1.0 (0.52–2.13)	0.869	1.3 (0.58–2.90)	0.522
Have you received any specific training related to infectious disease (including Mpox)?	No	73 (40.6)	107 (59.4)	1	1	1	
	Yes	115 (56.9)	87 (43.1)	1.9 (1.28–2.91)	0.001	1.9 (1.18–3.07)	0.008^*^
Have you had any direct experience managing or treating patients with infectious diseases (e.g. COVID-19, smallpox, etc.)?	No	62 (41.3)	88 (58.7)	1	1	1	
	Yes	126 (54.3)	106 (45.7)	1.6 (1.11–2.55)	0.014	1.32 (0.81–2.18)	0.26

*Significance at *p* < 0.05.

## 4 Discussion

Currently, there have been no reports of the Mpox outbreak in Ethiopia. However, the nation is vulnerable because of its shared border with Somalia and Sudan where cases have been documented. In addition to this, a recent report from Somalia Regional Health Office in Ethiopia indicates that individuals suspected of Mpox infection have been isolated (32). This highlights the potential risk for local transmission of Mpox from neighboring countries. This study reflects HCWs’ Mpox preparedness by examining the knowledge, attitudes, and associated factors influencing HCW’s readiness.

This study found that 48.4% of the HCWs had good knowledge. This is align with the study reported in Nigeria 52.2% (33) and Saudi Arabia 55.5% (34). Contrary to this, some studies reported a significant lower levels of knowledge among HCWs, for instance, Cameron (35), Turkey (36), Indonesia (31), Lebanon (29), and Pakistan (37) where 42.1, 32.5, 36.5, 33.7, and 34.4% of HCWs, respectively, had good knowledge about Mpox. The reason for this variation might be such as the accessibility and availability of training programs, sociocultural differences, and educational curriculum differences between countries. HCWs’ knowledge ratings may be better in areas with strong health education programs and continuous public health campaigns (38). Furthermore, variations in the local prevalence of Mpox and the unique characteristics of the healthcare system may have an additional impact on HCW’s knowledge and awareness of this illness.

Additionally, the study identified variables that are significantly associated with HCW’s knowledge and attitude. Notably, HCWs aged ≥50 Years old were 4.1 times more likely to have good knowledge of Mpox compared to younger. This might be due to those older HCWs especially those over 50, having more experience with outbreaks and health crises. They’ve likely had more training on infectious diseases, including Mpox. Their longer experience working in health institutions also helps to increase awareness of public health issues. Due to this and various reasons older HCWs have higher Mpox knowledge levels than younger. Furthermore, our study showed that Physicians and pharmacists were more likely to possess a good knowledge of Mpox compared to nurses. This aligns with the finding from Awoyomi et al., who identified a significant association between occupation and good knowledge among their respondents (39). The reason might be due to their specialized training, education curriculum, and job description. A physician mainly focuses on diagnostics and comprehensive disease management, including Mpox. Additionally, during these kinds of endemics, Physicians may have the chance to attend conferences that provide them with up-to-date information on the status of global disease knowledge. This was addressed by a study done in Indonesia that indicated Physician has more knowledge and confidence when they attend at least one national conference (40). While pharmacy professionals majorly focus on drug management and therapeutic intervention, stay updated on Mpox management. On the other hand, nurses place a higher priority on providing direct patient care, which would restrict their exposure to Mpox complex pharmacological and diagnostic features. These variations demonstrate how role-specific training and duties lead to variations in knowledge within healthcare occupations (41). This finding is in line with other studies which showed physicians had good knowledge of Mpox conducted in Peru (42), Lebanon (29), Kuwait (43), and Jordan (30). However, Alshahrani et al. found that 55% of Saudi Arabian doctors knew a good deal about Mpox, which the authors deemed to be a low level (34).

In our study, HCWs with a B.Sc., M.Sc., or higher qualifications were strongly associated with good knowledge of Mpox than having a Diploma or Certificate, aligning with a study previously conducted in China (44), Bangladesh (45), and Nigeria (39). This may be attributed to higher education equipping healthcare workers with more comprehensive knowledge and a better understanding of Mpox. After all, it exposes them to more complex health conditions and provides deeper training (46). Higher education programs enhance critical thinking and evidence-based practice to help HCWs better use their Mpox knowledge (47). Encouraging greater education may enhance responses to infectious diseases (48). Alongside this, our study showed a strong correlation between HCW’s Mpox knowledge and their years of work experience as well as their involvement in pertinent training, such as Mpox. Our results were supported by earlier studies conducted in Saudi Arabia (49), Indonesia (40), and Italy (50). These studies found that attending medical training was significantly associated with having good knowledge about Mpox, and that HCWs knowledge level increased with prolonged education and training in healthcare centers. This is because proper training and ongoing medical education are crucial for ensuring the development of confidence in diagnosing and treating infectious diseases (51).

In the attitude scale, this study revealed that 49.20% of the HCWs showed a positive attitude towards Mpox which was comparable with a study conducted in Nepale (52). The results of our study were superior to those of a study carried out in Turkey (36) and Pakistan (37). The shown discrepancy may be due to cultural and geographic disparities since areas with higher illness frequency or more media coverage tend to have more positive attitudes towards Mpox. Other possible reasons may be due to differences in healthcare education and training may be a factor in these discrepancies. Besides this, variations in survey techniques, sample sizes, and regional Mpox health regulations may potentially affect attitude assessments (53). Furthermore, the positive attitude of the healthcare workers in our study may have been influenced by their prior experience with the COVID-19 pandemic, which made them aware of its severity and receptive to learning more about the virus.

In our study, occupation, educational status, work experience, and training related to infectious disease were significantly associated variables with Mpox infection. Which is in line with earlier research showing that proactive attitudes and awareness of Mpox and other zoonotic illnesses are positively correlated with higher educational level and occupation (54, 55). However, the findings of our study are not consistent with those of Nepalese HCWs (52), who showed that attitudes regarding Mpox were not substantially correlated with occupation and educational status. This discrepancy raises the possibility that cultural attitudes, perceived disease danger, and systemic support aside from formal education and training may affect how healthcare professionals react to newly emerging diseases in various contexts. A proactive approach to infectious diseases is often associated with considerable training, but its impact may be lessened in situations where public health is not given as much priority or if disease exposure is low, according to other studies (56).

This study aimed to explore the knowledge and attitudes of healthcare workers about Mpox. However, one key limitation is that the cross-sectional design only offers a snapshot of data at a single moment in time, making it difficult to determine cause-and-effect relationships. To better understand these connections, future research could consider using cohort or case study designs, which would provide richer and more meaningful insights.

## 5 Conclusion

Based on our study HCWs’ knowledge and attitudes toward Mpox infection are relatively low. Variables such as occupation, educational status, work experience, and infectious disease training are significantly associated with HCWs knowledge and attitudes toward Mpox. However, with nearly half of the participants showing limited knowledge and negative attitudes, it becomes evident that there is a significant need for enhanced preparedness. This is crucial to ensure effective responses to future outbreaks of infectious diseases. Addressing this gap requires a commitment to supporting HCWs training initiatives, integrating infectious disease education into ongoing professional development programs, and ensuring that resources are more readily accessible.

## Data Availability

The original contributions presented in the study are included in the article/Supplementary material, further inquiries can be directed to the corresponding author.
