# Supplementary material for: Assessment of healthcare workers knowledge and attitudes towards Mpox infection at University of Gondar Comprehensive Specialized Referral Hospital, Ethiopia
Source: Front Public Health. 2025 Mar 3;13:1527315. doi: 10.3389/fpubh.2025.1527315 (PMC11911357; doi:10.3389/fpubh.2025.1527315)
Supplement: Supplementary file 1 [file Data_Sheet_1.docx]

**ANNEX I**

Annex I: Research participant information sheet and consent form

Gondar University

College of medicine and health science

School of pharmacy

Questionnaire for data collection on **“Knowledge, Attitude and Associated Factors of Monkeypox Infection among Healthcare Workers in university of Gondar comprehensive specialized Hospital”**

Code NO. ___________

Consent Form

Hello, my name is Alemante Tafese, I am lecturer at Gondar University College of medicine and health science school of pharmacy, this study is to examine health professional knowledge and attitude of Monkeypox among Healthcare Workers in university of Gondar comprehensive specialized Hospital.

I would like to ask few questions regarding to Knowledge and attitude regarding to Monkypox. The interview would take maximum of 25 minute of your time. The result will disseminate to concerned body to show gap and take interventions. Involvement in this study is voluntary and you may choose to participate or not to participate. Please feel free to ask any questions that you may have about the research; I will be glad to explain anything in greater detail.

All information will be kept anonymous and confidential. There will be no information that will identify you in particular. The findings of the study will be general for the study community and will not reflect anything particular to individual persons. The questionnaire will be coded to conceal identity.

There is no risk imposed on you by participating in this study. If you do not wish to continue, you have the right to withdraw from the study, without penalty, at any time. No any incentive that you get by participating in this study. If there are any questions or enquires any time about the study or the procedures, please contact: 0918237517, alemante45@gmail.com

Are you willing to participate? Yes

No

**1. Socio-demographic Characteristics of responder**

|  | **1. Socio-demographic Characteristics of responder** | |  |
| --- | --- | --- | --- |
| 1 | Age | ____________ |  |
| 2 | Gender | Male___________ |  |
|  |  | Female___________ |  |
| 3 | Residency | Urban__________ |  |
|  |  | Rural_________ |  |
|  | Level of education | Diploma |  |
|  |  | Degree |  |
|  |  | MSc and above |  |
| 4. | Monthly income in ETB | < 5000______ |  |
|  |  | 5000–8000_____ |  |
|  |  | 8000–12000_____ |  |
|  |  | >12000______ |  |
| 5. | Occupational status | Physician _____ |  |
|  |  | Pharmacist _____ |  |
|  |  | Nurse ______ |  |
|  |  | Other ( midwife , laboratory, dentist ) _____ |  |
| 7. | Work experience | <3 years_______ |  |
|  |  | 3–5 years______ |  |
|  |  | 5–10 years_____ |  |
|  |  | ≥10 years______ |  |
| 6 | Being vaccinated against COVID-19 | Yes |  |
|  |  | No |  |
| 7 | Source of information | Medical book or during study |  |
|  |  | Collogues |  |
|  |  | Social media |  |
|  |  | Tv,radio or mainstreem midia |  |

**2. The knowledge scale items’ responses.**

|  | **Items** | **“Yes”** | **“don’t know”** | **“No”** |
| --- | --- | --- | --- | --- |
| **1** | MPOX is prevalent in the Middle East |  |  |  |
| **2** | MPOX is prevalent in Western and Central Africa |  |  |  |
| **3** | There is an outbreak of human MPOX in the world |  |  |  |
| **4** | MPOX is caused by a virus |  |  |  |
| **5** | Human-to-human transmission of MPOX occurs through  skin-to-skin contact |  |  |  |
| **6** | Human-to-human transmission of MPOX occurs through touching objects or surfaces that have been used by someone with MPOX |  |  |  |
| **7.** | Human-to-human transmission of MPOX occurs through contact with respiratory secretions |  |  |  |
| **8.** | MPOX and smallpox have similar signs and symptoms |  |  |  |
| **9.** | Skin rash is one of the signs or symptoms of human MPOX |  |  |  |
| **10.** | Pustule is one of the signs or symptoms of human MPOX |  |  |  |
| **11.** | Antibiotics are used to treat human MPOX |  |  |  |
| **12.** | Diarrhea is one of the signs or symptoms of human MPOX |  |  |  |
| **13.** | Vaccination is available to prevent human MPOX |  |  |  |
| **14** | Young children less than 8 years of age are at increased risk for severe MPOX disease |  |  |  |
| **15.** | Pregnant women are at increased risk for severe MPOX disease |  |  |  |
| **16.** | Immune-compromised patients are at increased risk for severe MPOX disease |  |  |  |
| **17.** | Individuals with a history of atopic dermatitis or eczema are at increased risk for severe MPOX disease |  |  |  |

**1.2 Monkey pox Attitude Scale Items**

|  |  | Strongly Disagree | Disagree | Neutral | Agree | Strongly agree |
| --- | --- | --- | --- | --- | --- | --- |
| 1 | In my opinion, early detection of MPOX virus  can improve treatment and outcome |  |  |  |  |  |
| 2. | In my opinion, MPOX virus can be treated  at home |  |  |  |  |  |
| 3. | In my opinion, the MPOX virus transmission  can be reduced by following the appropriate  instructions provided |  |  |  |  |  |
| 4. | In my opinion, if there is an available MPOX  vaccine for the disease, it should be used |  |  |  |  |  |
| 5. | In my opinion, awareness of MPOX disease in  society is sufficient |  |  |  |  |  |
| 6. | In my opinion, MPOX can cause death |  |  |  |  |  |
| 7. | In my opinion, MPOX virus can be transmitted  from domestic pets to humans |  |  |  |  |  |
| 8. | In my opinion, the authorities should restrict travel to and from the areas of outbreak of MPOX disease |  |  |  |  |  |
| 9. | In my opinion, authorities should isolate  people infected with MPOX in  isolate hospitals |  |  |  |  |  |
